# Passive Immunity Establishment Through Colostral IgG Absorption in Neonatal Ruminants: Foundation for Efficient Ruminant Production

**DOI:** 10.3390/ani15213093

**Published:** 2025-10-24

**Authors:** Chao Yang, Mei Du, Anum Ali Ahmad, Yan Cheng, Kefyalew Gebeyew

**Affiliations:** 1 State Key Laboratory of Plateau Ecology and Agriculture, Qinghai University, Xining 810016, China; 2 College of Agriculture and Animal Husbandry, Qinghai University, Xining 810016, China; 3 The Roslin Institute, The University of Edinburgh, Easter Bush Campus, Edinburgh EH25 9RG, UK; 4 College of Life and Environmental Sciences, Hunan University of Arts and Science, Changde 415000, China; 5 Institute of Subtropical Agriculture, The Chinese Academy of Sciences, Changsha 410125, China

**Keywords:** neonatal ruminants, passive immunity, IgG absorption, colostrum, efficient production

## Abstract

**Simple Summary:**

Young animals are fundamental to sustainable animal husbandry, yet the management and feeding strategies for young ruminants face significant challenges, particularly high illness and mortality rates in early development. Neonatal hypogammaglobulinemia, a primary cause of this, results from the epitheliochorial placental structure preventing prenatal maternal immunoglobulin transfer. Consequently, neonatal ruminants must acquire essential passive immunity—defined as specific immune protection through passive reception of antibodies—solely via timely colostrum consumption. This review synthesizes current knowledge on the pathways, molecular mechanisms, and influencing factors of passive immunity establishment in neonatal ruminants, alongside its long-term impact on adult production performance. A comprehensive understanding of these aspects provides a scientific basis for optimizing colostrum feeding strategies and guiding future research into IgG absorption mechanisms, ultimately enhancing ruminant health and productivity.

**Abstract:**

Passive immunity, the acquisition of specific immune protection through external antibodies or immune components, is critically important for neonatal survival. In ruminants, however, neonatal hypogammaglobulinemia, a consequence of their epitheliochorial placental structure preventing prenatal antibody transfer, often leads to high morbidity and mortality. Consequently, neonatal ruminants are entirely dependent on the timely consumption of colostrum to acquire sufficient immunoglobulin G (IgG) for protection. Establishing robust passive immunity is therefore a cornerstone for their survival, healthy development, and future production efficiency. This review synthesizes current knowledge on the establishment of passive immunity in neonatal ruminants. We first outline the fundamental principles of passive immunity transfer, then delve into the specific pathways and molecular mechanisms in ruminants. Key factors influencing this process are subsequently discussed. Furthermore, we highlight the long-term impact of passive immunity on adult production performance. This review aims to provide a scientific foundation for optimizing colostrum management strategies and to stimulate future research into the intricate mechanisms of IgG absorption.

## 1. Introduction

Young animals are the cornerstone of sustainable animal husbandry, with their health directly impacting the industry’s long-term productivity and economic viability [1]. However, suboptimal management and feeding strategies for young ruminants often lead to significant challenges, primarily characterized by high morbidity and mortality rates during their early developmental stages. Globally, perinatal mortality (birth to 48 h post-birth) in dairy calves ranges from 3% to 9% [2]. Pre-weaning mortality rates are also substantial, reaching 9% in commercial dairy goat farms [3] and ranging from 6% to 14% in neonatal lambs [4]. In China, mortality rates for young livestock in small and medium-sized dairy and beef cattle farms, as well as sheep farms or individual households, are even higher, ranging from 10% to 30% [5]. Such high postnatal mortality significantly reduces farm income globally and severely restricts the high-quality and efficient development of the ruminant production industry [6]. Major contributors to morbidity and mortality in beef and dairy calves include diarrhea and pneumonia [7,8]. Similarly, septicaemia, pneumonia, and gastrointestinal infections are primary causes of high mortality in newborn lambs [4] and goats [3]. Crucially, failure of passive transfer (FPT) of immunity during the neonatal stage is a major contributing factor to young animal mortality, accounting for 31–39% of calf deaths [9]. Consequently, the critical role of adequate passive immunity in mitigating morbidity, mortality, and ensuring subsequent growth and welfare of newborn ruminants is internationally acknowledged.

Passive immunity, defined as the acquisition of specific immune protection through the passive reception of antibodies, lymphocytes, or other immune components [10], is essential for neonatal survival. Due to their unique epitheliochorial placental structure, ruminants prevent the prenatal transfer of maternal immunoglobulins to the fetus, resulting in neonates being born agammaglobulinemic [11]. Therefore, the establishment of passive immunity in newborn ruminants is entirely dependent on the timely absorption of an adequate amount of maternal immunoglobulin G (IgG) from colostrum through the small intestine, primarily within the first 24 h after birth [9]. This process, often referred to as ‘passive transfer of immunity’, is more accurately termed ‘transfer of passive immunity’ [12]. The successful establishment of this early passive immunity confers significant long-term benefits, including improved growth performance and reproductive efficiency [13]. The main pathway for establishment of passive immunity in newborn ruminants is the timely absorption of an adequate amount of immunoglobulin G (IgG) from the maternal colostrum shortly after birth.

The intestinal absorption of maternal immunoglobulins, particularly IgG, from colostrum is paramount for successful passive transfer in neonatal ruminants. Historically, successful passive transfer in dairy calves was defined by serum IgG concentrations of ≥10 mg/mL at 24 to 48 h post-birth, with values below this indicating failure of passive transfer [14]. However, advancements in research have led to new recommendations, categorizing neonatal calf serum IgG into four levels: excellent (≥25.0 g/L), good (18.0–24.9 g/L), fair (10.0–17.9 g/L), and poor (<10 g/L) [12]. In contrast, comparable precise information for dairy goats and lambs is limited, and reported serum IgG thresholds in the literature are often arbitrary and lack robust justification. For instance, a previous study suggested that blood IgG levels of 8 g/L could be protective for survival in kids reared in extensive systems [15], while another study demonstrated that serum IgG levels should exceed 12 g/L to ensure good health and pre-weaning survival in dairy goat kids under intensive management conditions [16]. More recently, a study reported that IgG concentrations lower than 11.4 g/L were strong indicators of increased early mortality risks in dairy goat kids [3]. Despite these efforts to establish thresholds for passive immunity transfer in dairy goat kids, these values remain less precise and standardized compared to those established for neonatal calves.

The efficiency of colostrum-derived IgG absorption in newborn ruminants is critically influenced by various factors, including colostrum quality, feeding quantity, and feeding time [17,18,19]. Despite this, current feeding strategies and management practices for newborn ruminants often lack standardized guidelines for establishing passive immunity, contributing to a high mortality rate associated with failure of passive transfer. Furthermore, the precise molecular mechanisms underlying colostral IgG absorption in neonatal ruminants remain largely unclear.

Therefore, this review aims to comprehensively synthesize the scientific literature on the different forms of passive immunity in mammals, the molecular mechanisms of IgG absorption, the factors affecting passive immunity establishment, and the relationship between passive immunity and efficient production in ruminants. Ultimately, this review seeks to provide a scientific basis for optimizing passive immunity success rates in newborn ruminants and fostering the high-quality development of the ruminant livestock industry.

## 2. Passive Immunity Transfer in Neonatal Animals

Passive immunity is broadly categorized into natural passive immunity and artificial passive immunity [20]. Natural passive immunity involves the transfer of maternal immunoglobulins to the offspring via the placenta, yolk sac, or colostrum, thereby contributing to the establishment of offspring immunity [21]. Conversely, artificial passive immunity entails the exogenous administration of specific antibodies to confer immediate protection to immunologically naive individuals [22]. For neonatal animals, natural passive immunity, primarily through the transfer of maternal immunoglobulins, represents the predominant mechanism for acquiring immune protection [10]. The specific routes of passive immunity transfer exhibit considerable interspecies variation, largely dictated by the maternal placental structure [23]. In species with hemochorial placentas, such as primates, and in some other mammalian species, immunoglobulins are transferred transplacentally to the fetus during gestation [24]. Rodents, including rats and mice, demonstrate both prenatal (transplacental) and postnatal (via milk) immunoglobulin transfer [25,26]. Notably, ruminants possess a specialized synepitheliochorial placenta, characterized by multiple tissue layers separating maternal and fetal circulation. This structural impediment effectively precludes the prenatal transfer of maternal immunoglobulins to the fetus [27]. These observations underscore that the mode of passive immunity acquisition in ruminants is distinctly different from that in many other mammalian species. Irrespective of the specific pathway, passive immunity is indispensable for the establishment of robust systemic and mucosal immunity in neonatal mammals.

### 2.1. Passive Immunity Transfer and Systemic Immunity

The structure of maternal immunoglobulins, as the main antibodies in the body, is thought to be separated functionally into variable domains that bind antigens and constant domains that specify effector functions [28]. Thus, maternal antibody transfer is an important mechanism for protecting newborns while their immune systems are imperfect [29]. The systemic immunity (primarily through the passive transfer of immunoglobulins) varies among species [30]. It generally occurs in three forms of passive immunoglobulins transfer: prenatal transfer only, combined prenatal and postnatal transfer, and postnatal transfer only.

#### 2.1.1. Passive Immunity Transfer During the Pre-Natal Period

In species possessing a hemochorial placenta, such as primates, rabbits, and guinea pigs, trophoblast cells extensively invade maternal tissue, establishing direct contact between the chorionic surface and maternal circulation [31]. This unique placental architecture facilitates the prenatal transfer of maternal immunoglobulins, predominantly IgG, to the fetus [24,32]. Specifically, in primates, IgG transfer occurs almost entirely transplacentally, a process mediated by receptor-mediated endocytosis involving extravillous trophoblast and fetal endothelial cells [33]. The human placenta, for instance, efficiently transfers a wide array of pathogen-specific protective IgG antibodies, including those against hepatitis B, measles, and group B streptococcus. This efficient transfer provides a crucial foundation for neonatal immunotherapy strategies, such as maternal vaccination aimed at enhancing newborn protection. In contrast, rabbits and guinea pigs primarily achieve prenatal IgG transfer via the yolk sac [34]. Collectively, the hemochorial placental structure in these species ensures effective maternal-fetal IgG transfer during gestation, conferring essential passive immunity to the developing offspring.

#### 2.1.2. Passive Immunity Transfer in Both Pre- and Post-Natal Periods

The endotheliochorial placenta, found in rats, mice, cats, and dogs, is characterized by uterine endothelial cells in direct contact with chorionic villi [35]. Its trophoblast layer, composed of cytotrophoblasts and syncytiotrophoblasts, exhibits moderate invasion of the maternal tissue [36]. In this placental type, fetal and maternal blood circulation follows a simple cross-flow pattern, where fetal blood flows horizontally across maternal blood [37]. In these animals, passive transfer of immunoglobulins occurs both pre- and postnatally, primarily through the yolk sac/placenta and the fetal intestine after birth. During the prenatal stage, IgG can rapidly bind to receptors present on the surface of the yolk sac membrane, be internalized in clathrin-coated vesicles, and be stored in apical vesicles early in pregnancy. Later, IgG is either hydrolyzed or transferred into the fetal capillaries [38]. In rodents, although some IgG crosses the placenta prenatally, the amount is small. The majority of IgG that establishes passive immunity is absorbed from colostrum postnatally through FcRn receptor-mediated transport pathway in the intestine, with absorption occurring predominantly within the first three days [39].

#### 2.1.3. Passive Immunity Transfer During the Postnatal Period

In ruminants, including cattle and sheep, the placenta is characterized by its cotyledonary (synepitheliochorial) structure, featuring multiple independent placental units (cotyledons) [40]. The intercotyledonary regions consist of the smooth chorionic allantois. This placental type is composed of six cellular layers, forming a robust placental barrier that effectively precludes the prenatal transfer of maternal antibodies to the fetus, consequently leading to neonatal agammaglobulinemia [41]. Therefore, the acquisition of passive immunity in these species is exclusively postnatal, primarily through the timely consumption of colostrum, which provides essential IgG. Similarly, species with a diffuse epitheliochorial placenta, such as horses, donkeys, pigs, and llamas, also exhibit a non-invasive trophoblast layer that attaches or fuses with the uterine surface epithelium without invading maternal tissue [42]. While the uterine mucosal stroma in these species primarily enhances blood flow to deliver nutrients and promote local angiogenesis during pregnancy, the fundamental placental structure remains largely unchanged. Despite extensive interdigitation of fetal and maternal tissues, which creates a large contact surface, this placental barrier, also comprising six cell layers, effectively separates fetal and maternal circulations. This structural arrangement prevents the passage of large molecules like immunoglobulins throughout gestation [43]. These collective observations underscore that in all species with an epitheliochorial placenta, including ruminants, horses, and pigs, the impermeable placental barrier necessitates the postnatal acquisition of passive immunity exclusively via colostrum.

### 2.2. Passive Immunity Transfer and Local Mucosal Immunity

Maternal colostrum and milk are pivotal for providing passive protection to the mucosal surfaces of neonatal animals [44]. This protective effect is mediated by both specific immune components and non-specific factors, including lactoferrin, lysozyme, fatty acids, and complement proteins [45]. Unlike IgG, other protective antibodies present in colostrum and milk, such as IgA, IgE, and IgM, are almost unable to be absorbed or only minimally absorbed by neonatal animals [32,46,47]. Instead, these immunoglobulins exert their protective effects locally within the gastrointestinal tract, where they neutralize viruses or virulence factors, bind to microbial pathogens, and prevent their attachment to mucosal surfaces, thereby conferring essential local mucosal immunity [48,49].

Immunoglobulin profiles in colostrum exhibit significant interspecies variation. In ruminants, IgG is the predominant immunoglobulin in colostrum (Table 1). However, for sustained protection against gastrointestinal infections like rotavirus and coronavirus, the continuous presence of specific IgA antibodies from mature milk in the intestinal lumen is critical in calves [50]. In pigs and horses, while IgG dominates in colostrum, it is gradually superseded by IgA as colostrum transitions to mature milk [51]. Notably, studies indicate that piglets receiving IgA generally exhibit stronger passive resistance to enteric infections, such as gastroenteritis virus infection, compared to those primarily receiving IgG [52]. In contrast, human neonates acquire IgG prenatally, and IgA is the predominant immunoglobulin in human colostrum (Table 1) [53]. Functionally, IgA primarily operates as secretory IgA (sIgA), which plays a crucial role in mucosal protection by adhering to pathogens and preventing their colonization. The secretory component of sIgA enhances its resistance to proteolytic enzymes and gastric acid, thereby improving antibody stability and efficacy in the harsh gastrointestinal environment [54]. Beyond providing passive mucosal immunity, colostrum also stimulates the secretion of sIgA in the goat’s digestive tract, thereby accelerating the establishment of their active immune defenses [55]. In summary, while IgG is the primary immunoglobulin for systemic passive immunity in many species, IgA, particularly in its sIgA form, is indispensable for local mucosal protection in neonatal mammals. Although IgA levels in the colostrum of ruminants, pigs, and horses may be lower than IgG, its role in establishing local mucosal immunity is significant. In humans, where prenatal IgG transfer is complete, IgA is the main immunoglobulin in colostrum, actively preventing pathogen colonization and contributing to the development of the neonate’s own active immune responses.

## 3. Pathways and Molecular Mechanisms of Passive Immunity Establishment in Neonatal Ruminants

The establishment of passive immunity in neonatal ruminants is critically dependent on the timely ingestion of adequate colostral immunoglobulins, primarily IgG, post-natally. The absorption of these colostrum-derived IgG predominantly occurs in the small intestine of neonatal ruminants [61]. However, the precise mechanism underlying this process—whether it is a specific, receptor-mediated event or a non-specific uptake—remains a subject of ongoing debate. Historically, it was widely hypothesized that intestinal IgG absorption proceeded via non-specific pinocytosis. In this model, enterocytes were thought to internalize IgG molecules from the intestinal lumen through fluid-phase endocytosis, subsequently transporting them across the cell and releasing them into the neonatal circulation [62]. In contrast, more recent evidence suggests the involvement of the neonatal Fc receptor (FcRn), which is expressed in the small intestine of neonatal ruminants and actively mediates IgG absorption from colostrum [63]. The synthesis of FcRn initiates in the rough endoplasmic reticulum, followed by processing in the cis-Golgi network, and eventual trafficking to and incorporation into the apical membrane domain of enterocytes [64]. During the neonatal period, the initial step of IgG absorption involves FcRn binding to IgG within the acidic pH environment of the intestinal lumen. Subsequently, the FcRn-IgG complex is internalized into coated vesicles and translocated through endosomes within the enterocytes [65]. Upon reaching the basolateral membrane, the FcRn-IgG complex dissociates in the neutral pH environment, releasing IgG into the intercellular space, from where it enters the bloodstream [66]. Concurrently, the FcRn-containing endosome undergoes basolateral transcytosis, recycling back to the apical membrane domain for further IgG uptake [67]. Despite the established role of FcRn, previous studies have reported an increase in the expression of the FCGRT gene, encoding the α heavy chain of FcRn, with developmental time in goat kids [68]. This observation appears contradictory, as the overall capacity for IgG absorption significantly decreases with advancing age in neonatal ruminants. This discrepancy suggests that FcRn expression alone may not be the sole or primary determinant regulating the efficiency of IgG absorption in neonatal ruminants. Further support for this notion comes from studies in Caco-2 cells, where relative *FCGRT* mRNA expression increased with culture duration, yet validation experiments indicated that FcRn primarily facilitated IgG uptake rather than its subsequent transcellular transport [69]. Otherwise, single-nucleotide polymorphisms in *FCGRT* showed an association with serum IgG concentration in newborn calves [70]. Therefore, the observed decline in IgG absorption capacity with developmental time may be more accurately attributed to dynamic changes in the overall endocytic and transcytotic machinery, rather than solely to FcRn expression levels.

**Figure 1:**
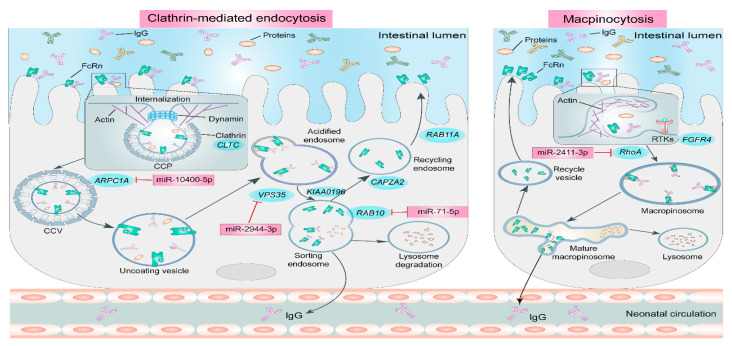
Mechanism of delayed colostrum feeding affecting IgG absorption in neonatal goats [68].

In neonatal goats, the efficiency of colostral IgG absorption significantly declines when feeding occurs beyond 24 h post-partum. This reduction in absorptive capacity has been directly linked to a decrease in the activity of both clathrin-mediated endocytic and macropinocytic pathways within the jejunum [68]. Specifically, multi-omics analyses revealed the downregulation of genes critical for these processes. These include genes involved in clathrin synthesis (*CLTC*), actin-related clathrin-coated vesicle formation (*ARPC1A*), sorting and recycling endosomes (*CAPZA2*, *KIAA0196*, *RAB10*, *RAB11A*, and *VPS35*), and macropinosome formation (*FGFR4* and *RhoA*). Such widespread downregulation compromises the efficiency of both IgG internalization and subsequent transcytosis. Furthermore, several microRNAs (miRNAs), including miR-2755-3p, miR-10400-5p, miR-2944-3p, miR-71-5p, and miR-2411-3p, have been identified as negative regulators of clathrin-coated vesicle formation, FcRn-IgG complex sorting, FcRn recycling, and macropinosome formation (Figure 1). Collectively, these findings strongly suggest that the observed loss of IgG absorption capacity after 24 h post-partum is primarily attributable to the diminished activity of clathrin-mediated endocytosis and macropinocytosis. While these compelling evidences are derived from multi-omics analyses, the precise functions of the identified mRNAs and miRNAs in regulating clathrin-mediated endocytosis and macropinocytosis require further validation at the primary cell level. Moreover, research focusing on the molecular mechanisms of IgG absorption in other ruminant species, such as sheep and calves, remains notably limited.

## 4. Factors Influencing the Establishment of Passive Immunity in Neonatal Ruminants

In neonatal ruminants, the efficiency of IgG absorption is critically influenced by several key determinants, including colostrum quality, feeding volume, and the timing of colostrum administration [71]. Research in this area has predominantly focused on calves and lambs, primarily due to the epitheliochorial placental structure characteristic of ruminants, which precludes prenatal maternal IgG transfer. Consequently, postnatal colostrum intake represents the exclusive pathway for the establishment of passive immunity. Building upon this foundation, this review comprehensively examines the impact of colostrum quality, feeding volume, feeding time, and hygienic practices on IgG absorption in neonatal ruminants.

### 4.1. Colostrum Quality

IgG constitutes over 85% of the total immunoglobulins in ruminant colostrum, making its concentration a critical determinant of colostrum quality. A concentration exceeding 50 g/L is widely recognized as indicative of high-quality colostrum [72]. Practically, colostral IgG content exhibits substantial individual variability, with an average concentration of 76 g/L reported, and a wide range from 9 to 186 g/L observed in Holstein dairy cows [73]. This variability in colostrum quality is influenced by several factors, including breed, dam’s age, parity, heat stress, and the timing of colostrum collection [74]. Numerous studies underscore the positive correlation between colostral IgG concentration and passive immunity establishment. For instance, calves fed 4 L of colostrum (average IgG: 70.4 g/L, collected immediately post-calving), transitional milk (average IgG: 38.6 g/L, collected 48–72 h post-calving), or bulk tank milk (average IgG: 0.6 g/L) demonstrated serum IgG concentrations at 24 and 48 h that were directly proportional to the ingested IgG quality. Furthermore, maximum IgG absorption efficiency significantly increased with improved colostral IgG concentration [75]. Similarly, calves administered 3.8 L of colostrum containing 30 g/L, 60 g/L, or 90 g/L IgG within 2 h of birth exhibited a significant dose-dependent increase in serum IgG levels from 4 to 48 h, with the success rate of passive immunity rising proportionally with colostral IgG content [72]. In Jersey calves, a significant reduction in both total protein and IgG concentrations in serum was observed when equal volumes of colostrum with lower IgG concentrations (31.2 mg/mL) were fed compared to higher concentrations (84 mg/mL) [76]. Consistent with these findings, calves fed high-quality colostrum from their own dam (average IgG: 184.4 g/L) within 4 h of birth showed significantly higher serum IgG concentrations on days 1, 2, 3, and 7 compared to those fed pooled colostrum with a lower average IgG concentration (90.5 g/L) [77]. However, while high colostral IgG concentrations generally lead to higher rates of passive transfer, the apparent absorption efficacy can present a nuanced picture. For example, calves fed colostrum with a high IgG concentration (123.0 mg/mL) achieved higher rates of passive transfer compared to those fed adequate-quality colostrum (85.2 mg/mL), with both groups exceeding the threshold of 23 mg/mL serum IgG. Intriguingly, the apparent efficiency of absorption of IgG (AEA) was lower in calves fed high-quality colostrum (24.9%) compared to those fed adequate-quality colostrum (29.3%) [19]. This particular finding suggests that while feeding high-quality colostrum promotes robust passive immunity, neonatal calves may possess a limited capacity to absorb extremely high concentrations of IgG, potentially leading to a saturation effect on absorption efficiency. Therefore, these collective findings indicate that feeding high-quality colostrum post-natally is crucial for establishing passive immunity in calves, and that optimizing colostrum quality to ensure adequate, rather than excessively high, IgG concentrations may be beneficial for maximizing apparent absorption efficacy.

Evidence suggests that IgG absorption in neonatal lambs is a saturable process, leading to diminished absorption efficiency at high intake levels. This principle is demonstrated by several studies. For example, when lambs were administered ovine colostrum to provide IgG doses of 4 g/kg versus 8 g/kg BW, the higher dose failed to produce a proportionally greater increase in serum IgG concentration [78]. Similarly, another study compared the feeding of high-IgG bovine colostrum (115.8 mg/mL) with lower-IgG ovine colostrum (48.1 mg/mL) to newborn lambs [79]. Although lambs fed bovine colostrum achieved higher terminal serum IgG concentrations at 24 h post-birth, their AEA was significantly lower than that of lambs fed ovine colostrum (14.2% vs. 23.6%) [79]. Collectively, these findings indicate that the intestinal enterocytes of newborn lambs have a finite capacity for IgG transport. Once this capacity is exceeded, the rate of absorption becomes disproportional to the amount of IgG available in the intestinal lumen. Moreover, although bovine colostrum could be used to feed lambs, there was early cessation of absorption of bovine immunoglobulins by the gut of lambs [80].

The quality of colostrum extends beyond its immunoglobulin content to encompass its microbiological integrity. High bacterial loads, a common consequence of on-farm handling, can severely compromise the efficiency of passive transfer. This negative effect is attributed to several mechanisms, including bacterial–IgG binding, accelerated shedding of intestinal absorptive cells and disruption of IgG transport pathways [14]. Conversely, the taxonomic profile of the colostral microbiome can positively influence immune transfer. The presence of specific bacterial genera, including *Acinetobacter*, *Pseudomonas*, *Lactococcus*, and *Chryseobacterium*, has been correlated with elevated serum IgG concentrations and improved absorption efficiency in calves [81]. To control microbial contamination, pasteurization is frequently employed. While some protocols (60 °C for 30 min) have demonstrated a significant reduction in bacterial load and a corresponding 20–35% enhancement in IgG absorption efficiency [82], the efficacy of this practice remains a subject of debate. Contradictory findings show that other protocols (e.g., 60 °C for 60 min) yield no improvement, or even a slight reduction, in serum IgG levels [83]. A potential explanation for these conflicting results is that heat treatment itself can be detrimental, as exposure to 60 °C for 30–120 min may denature IgG, thereby reducing the total amount of functional antibody available for absorption [84]. Consequently, the application of heat treatment must be carefully calibrated to ensure that the benefits of pathogen reduction outweigh the potential risk of immunoglobulin degradation.

### 4.2. Colostrum Feeding Volume

The volume of colostrum administered is a critical determinant of IgG absorption, though its effect is modulated by IgG concentration. A dose-dependent relationship is often observed; for instance, increasing the feeding volume from 1.5 L to 2.5 L nearly doubled the serum IgG concentration in newborn calves [85]. Similarly, another study demonstrated that serum IgG levels in crossbred Holstein calves rose progressively as feeding volumes increased from 3 L to 4 L [86]. However, this linear relationship does not hold under all conditions. In a notable exception, Gamsjäger et al. reported [87] no significant difference in serum IgG concentrations between calves fed 1 L and 2 L of colostrum. This apparent contradiction can likely be explained by the concept of absorptive saturation. The colostrum used in that study had an exceptionally high IgG concentration (100 g/L), meaning the 1 L volume already delivered a massive dose (100 g) of IgG, which may have saturated the intestinal FcRn receptor system. In such cases, providing additional volume yields no further benefit. These findings underscore that the primary goal is to deliver a sufficient total mass of IgG. While industry guidelines often recommend a specific volume—such as 3 L of high-quality colostrum (>50 g/L IgG) within 2 h of birth, or a total of 4 L within 12 h [88], these should be viewed as practical benchmarks rather than rigid rules. The optimal feeding strategy is not based on volume alone, but on adjusting the volume according to colostrum quality to ensure a target IgG mass is ingested before gut closure.

To accurately assess the impact of colostrum feeding volume on IgG absorption, researchers provided newborn calves with colostrum at 7%, 8.5%, and 10% of body weight (BW), and serum IgG concentrations at 24, 48, and 72 h after birth were significantly higher in calves fed 8.5% BW colostrum compared to those fed 7% and 10% BW colostrum [89]. Interestingly, when colostrum IgG concentration exceeded 20 g/L, feeding 1 L resulted in greater absorption efficiency than feeding 2 L [90]. Additionally, calves receiving 2 L of colostrum immediately after birth, followed by another 2 L after 12 h, exhibited significantly higher serum IgG levels than calves that received a single 4 L of colostrum immediately after birth [76]. These findings suggest that immunoglobulin absorption efficiency is negatively correlated with the volume of colostrum fed in neonatal calves. When a larger volume of colostrum is provided, IgG absorption appears physiologically limited, possibly due to the transport capacity of IgG-like large molecules by the calf’s intestinal epithelium approaching saturation. However, colostrum feeding volume of sheep and goats lacks literature reports, few documents demonstrated that colostrum feeding volume of goats were 7.5% BW or 160 mL/kg BW [68,91]. In summary, overfeeding colostrum may inhibit the efficiency of IgG absorption in neonatal ruminants. Therefore, feeding a small volume of high-quality colostrum (with high IgG concentration) within a certain range is more beneficial for IgG absorption in neonates than feeding a large amount of low-quality colostrum (with low IgG concentration).

### 4.3. Colostrum Feeding Time

The efficacy of passive immunity transfer is critically dictated by the timing of the first colostrum feeding, a factor governed by the physiological process of intestinal closure. This process, defined as the time-dependent cessation of macromolecule (e.g., IgG) absorption by the intestinal epithelium, is not an abrupt event but a gradual decline. In neonatal ruminants, the capacity for IgG absorption is maximal at birth and declines precipitously thereafter, with the optimal window for efficient uptake occurring within the first 4–6 h of life and absorption becoming negligible by 24 h [92]. The profound impact of early feeding is well-documented in calves. For instance, providing colostrum immediately after birth, as opposed to delaying by 6 or 12 h, results in significantly higher serum IgG concentrations and greater AEA [17]. This principle is conserved across ruminant species. In goat kids, delaying the first meal from the initial 4 h window to 12–16 h post-birth severely impairs both serum IgG levels and apparent efficiency of absorption of IgG [93]. Furthermore, delayed colostrum feeding (44 h after birth) reduced serum IgG concentrations and the maximum apparent efficiency of absorption of black goat kids [68]. Similarly, lambs fed at 2 h post-birth achieve superior serum IgG concentrations compared to those first fed at 14 h [94]. Collectively, these studies provide unequivocal evidence that the timing of the first colostrum feeding is a paramount determinant of successful passive immunity. Administering colostrum within the first 2 h of life is critical to capitalize on the period of maximum intestinal permeability and ensure the establishment of robust immunity.

### 4.4. Other Influencing Factors

The absorptive environment of the neonatal gut can be actively manipulated through the supplementation of colostrum with exogenous compounds, leading to a spectrum of outcomes from beneficial to detrimental. For instance, interventions targeting gut health have produced conflicting results (Table 2). While adding probiotic *Lactobacillus* strains can improve IgG uptake [95], the prebiotic mannan oligosaccharide (MOS) has been shown to have the opposite effect, reducing IgG absorption in neonatal calves [96]. Yet, not all prebiotics are detrimental; difructose anhydride (DFA) III has been found to significantly enhance serum IgG and AEA, possibly by promoting transport through the paracellular pathway, a route distinct from standard endocytosis [97]. A similar variability is observed with plant-derived extracts. Capsaicin, for example, proved to be a potent enhancer of passive immunity, significantly increasing serum IgG when added to colostrum [98]. Conversely, green tea extract failed to produce any significant change in IgG absorption metrics [99]. These divergent findings strongly indicate that there is no universal benefit to supplementation. The effect of any given additive is highly dependent on its specific biochemical interaction with the intestinal epithelium. Therefore, a cautious, evidence-based approach is essential, as improper supplementation (e.g., with MOS) may inadvertently compromise, rather than enhance, the establishment of passive immunity.

The neonatal gut presents a complex environment where the integrity of ingested IgG must be preserved. Colostrum is naturally equipped with endogenous protease inhibitors to protect immunoglobulins from proteolytic degradation in the gastrointestinal tract. Attempts to augment this natural protection, for example, by supplementing colostrum with soybean trypsin inhibitor, have been shown to yield no additional benefit to serum IgG levels or AEA in goat kids [91]. This suggests that the protective capacity of high-quality colostrum is likely already sufficient, rendering further inhibition redundant. In contrast to redundancy, some supplements can cause direct interference. The addition of mannan oligosaccharides (MOS) to colostrum, for instance, resulted in significantly lower serum IgG concentrations in neonatal goats, particularly in the critical early hours post-birth. The proposed mechanism is physical: MOS may adhere to the intestinal wall, creating a barrier that sterically hinders IgG from reaching its transport receptors [100]. Therefore, a successful colostrum feeding strategy is not merely about adding beneficial components, but about preserving the inherent efficacy of colostrum and avoiding supplements that could inadvertently disrupt the delicate process of IgG absorption.

## 5. Colostrum Feeding, Passive Immunity Establishment and Efficient Production of Ruminants

The relationship between colostrum and the survival of newborn ruminants has been long characterized. During the neonatal period, adequate colostrum intake not only establishes passive immunity and reduces pre-weaning morbidity and mortality, but also enhances growth performance and exerts lasting effects on lifetime productivity. Dose-dependent effects have bene observed in calves were fed either 2 or 4 L of colostrum within 1 h of birth: those receiving 4 L exhibited a 0.28-pound higher average daily gain (ADG) over the first 80 days, a 30% higher growth rate before puberty, a 16% greater survival rate by the end of the second lactation, and a cumulative milk yield advantage of 2263 pound compared with calves fed 2 L [101]. An earlier age at first calving increased milk yield in the first and second lactations [102]. Restricting colostrum intake in neonatal calves decreases circulating concentrations of short- and long-chain acyl-carnitine, suggesting that mitochondrial function may be reprogrammed to support alternative energy-expenditure adaptations [103]. Feeding of pasteurized colostrum has been shown to reduce the incidence of bovine respiratory disease and diarrhea during the first year of life, increase calf body weight across the first three lactations, and enhance milk yield in the first lactation [104]. Thus, colostrum feeding strategies promote the growth and lactation performance, and reduce the incidence of diseases.

Beyond providing passive immunity, colostrum is a potent gut-trophic agent that actively drives the structural maturation of the neonatal intestine [105]. Its primary effect is the stimulation of mucosal growth, leading to a significant increase in villus height and overall absorptive surface area [106]. This is a dose-dependent response, with greater colostrum intake corresponding to larger villi. This rapid mucosal expansion is achieved through a dual-action mechanism. First, stimulation of cell proliferation: colostrum contains specific bioactive factors that stimulate the proliferation of crypt cells, which are the progenitors of the intestinal epithelium [107]. Second, inhibition of cell death: colostrum reduces the rate of apoptosis (programmed cell death) in existing enterocytes, thereby extending their functional lifespan [105]. Crucially, these developmental effects are independent of colostrum’s basic nutritional value. However, little information about the effects of colostrum ingestion on growth and reproductive performance, as well as the development of intestine in lambs and goats, was reported.

## 6. Conclusions and Perspectives

The successful establishment of passive immunity is a cornerstone of neonatal ruminant health, fundamentally influencing long-term growth, development, and survival. Decades of research have established a clear framework for best practices: the timely administration of a sufficient volume of high-quality, microbiologically clean colostrum is paramount. The efficacy of this process is a multifactorial equation, sensitive to IgG concentration (adequate colostrum quality), feeding volume (2–3 L), timing (0–2 h after birth), and the presence of modulatory additives. Despite this well-established practical knowledge, a significant gap persists between on-farm application and fundamental molecular understanding. The precise cellular mechanisms governing the time-dependent decline in IgG absorption—the process of intestinal closure—remain largely unresolved. While processes like clathrin-mediated endocytosis are implicated, the molecular choreography that dictates the kinetics of this closure is poorly defined. The neonatal Fc receptor (FcRn) is known to be the central orchestrator of IgG transport, yet the intricate details of its regulation in the neonatal gut are still being uncovered.

Future research must therefore prioritize the elucidation of these molecular pathways. A deeper understanding of FcRn regulation could unlock novel intervention strategies, such as the development of targeted supplements that prolong its expression or enhance its transport efficiency. Elucidating the mechanisms of gut closure could lead to methods for transiently and safely extending the window of absorption. Ultimately, transitioning from generalized recommendations to precision-guided colostrum management hinges on translating these molecular insights into evidence-based strategies that ensure every neonate achieves robust and lasting immunity.

## Figures and Tables

**Table 1:** Percentage of immunoglobulin in colostrum of Bovine, sheep, horses, pigs, and humans.

Species	Percentage of Immunoglobulin (%)			References
	IgA	IgG	IgM	
Bovine	5.0	88.0	7.0	Ahmann et al., 2021 [56]
Sheep	2.0	86.0	12.0	Klobasa and Werhahn, 1989 [57]
Horse	1.2	89.2	9.6	Hurley and Theil, 2011 [58]
Pig	14.9	77.7	7.4	Hăbeanu et al., 2022 [59]
Human	91.6	1.5	6.8	Akhter et al., 2022 [60]

**Table 2:** Effects of supplementation of exogenous probiotics, prebiotics, or plant extracts on IgG absorption in neonatal ruminants.

Species	Additives and Dosage	Colostrum Quantity and Quality	The Impact on IgG Absorption	References
Bovine	*Lactobacillus* strains, 1.85 × 10^7^ CFU	8% body weight (BW)	A significant increase in serum Ig G concentration was observed in the *Lactobacillus* strains supplemented groups (Treatment: 273.49 ± 6.36 ng/mL, CON:159.51 ± 5.10 ng/mL)	Saiady, 2010 [95]
	Mannan-oligosaccharide, 30 g	3.8 L, IgG concentration of 105.6 g/L	Supplementation of Mannan-oligosaccharide in colostrum decreased serum IgG level (Treatment: 24.02 ± 1.05 g/L, CON: 30.75 ± 1.04 g/L) and apparent efficiency of absorption of IgG (Treatment: 23.9 ± 0.97%, CON: 30.4 ± 0.96%)	Brady et al., 2015 [96]
	Green tea extract, 15 mL	3 L, IgG concentration of 50 g/L	Green tea extract of colostrum did not affect serum IgG concentration and apparent efficiency of absorption of IgG	Reis et al., 2022 [99]
	Capsaicin, 40 mg per kg of BW	10% BW in 1 h, 2 L on 12 h and 2 L on 20 h after birth, IgG concentration of 50.75 g/L	Supplementation of capsaicin in colostrum significantly increased serum IgG concentrations (Treatment: 21.6 ± 0.43 g/L, CON: 15.5 ± 0.78 g/L) than those only received equal volume of colostrum	Rodas et al., 2025 [98]
	Difructose anhydride (DFA) III, 18 g	2 L at 1, 10, and 24 h after birth, IgG concentration of 42.9 g/L	Supplementation of DFA III in colostrum increased serum IgG concentration on 10 h (Treatment: 13.8 ± 0.8 g/L, CON: 11.5 ± 0.9 g/L), 24 h (Treatment: 21.3 ± 1.5 g/L, CON: 17.5 ± 1.5 g/L), 36 h (Treatment: 22.3 ± 1.4 g/L, CON: 17.7 ± 1.4 g/L), day 4 (Treatment: 19.9 ± 1.2 g/L, CON: 15.7 ± 1.4 g/L) and day 7 (Treatment: 18.6 ± 1.1 g/L, CON: 14.5 ± 1.3 g/L) after birth, as well as apparent efficiency of absorption of IgG (Treatment: 36.1 ± 2.7%, CON: 29.5 ± 2.7%) at 36 h	Htun et al., 2016 [97]
Goat	Soybean trypsin inhibitor, 1 g/L colostrum	160 mL/kg BW, IgG concentration of 26.35 g/L	Supplemented soybean trypsin inhibitor in colostrum did not affect serum IgG levels at 24 h (Treatment: 13.7 ± 3.7 g/L, CON: 14.2 ± 3.7 g/L) and 48 h (Treatment: 12.6 ± 3.3 g/L, CON: 12.7 ± 3.6 g/L) and the apparent efficiency of absorption of IgG (Treatment: 25.2%, CON: 24.5%) in goat kids	Ramos et al., 2010 [91]
	Mannan oligosaccharides, 0.06% of birth BW	5% of BW colostrum, IgG concentration of 28.61 mg/mL	Supplemented mannan oligosaccharides in colostrum significantly decreased serum IgG concentration on 3 h and 6 h after birth	Yang et al., 2022 [100]

## Data Availability

Data sharing is not applicable to this article as no datasets were generated or analyzed during the current study.
